# Twinning of cubic diamond explains reported nanodiamond polymorphs

**DOI:** 10.1038/srep18381

**Published:** 2015-12-16

**Authors:** Péter Németh, Laurence A. J. Garvie, Peter R. Buseck

**Affiliations:** 1 Institute of Materials and Environmental Chemistry, Research Center for Natural Sciences, Hungarian Academy of Sciences, H-1117 Budapest, Magyar Tudósok Körútja 2, Hungary; 2 Center for Meteorite Studies, Arizona State University, Tempe, Arizona 85287-6004, USA; 3 School of Earth and Space Exploration, Arizona State University, Tempe, Arizona 85287-6004, USA; 4 School of Molecular Sciences, Arizona State University, Tempe, AZ 85287-1604, USA

## Abstract

The unusual physical properties and formation conditions attributed to h-, i-, m-, and n-nanodiamond polymorphs has resulted in their receiving much attention in the materials and planetary science literature. Their identification is based on diffraction features that are absent in ordinary cubic (c-) diamond (space group: Fd-3m). We show, using ultra-high-resolution transmission electron microscope (HRTEM) images of natural and synthetic nanodiamonds, that the diffraction features attributed to the reported polymorphs are consistent with c-diamond containing abundant defects. Combinations of {113} reflection and <011> rotation twins produce HRTEM images and d-spacings that match those attributed to h-, i-, and m-diamond. The diagnostic features of n-diamond in TEM images can arise from thickness effects of c-diamonds. Our data and interpretations strongly suggest that the reported nanodiamond polymorphs are in fact twinned c-diamond. We also report a new type of twin (<1
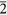
1> rotational), which can give rise to grains with dodecagonal symmetry. Our results show that twins are widespread in diamond nanocrystals. A high density of twins could strongly influence their applications.

Interest in diamond nanocrystals stems from applications that range from materials to medical sciences^1^,^2^,^3^,^4^,^5^. These nanocrystals possess high hardness and wear resistance, are chemically inert, and are promising for drug delivery. Wide band gap, optical transparency, and unusually high thermal conductivity make them attractive materials for preparing semiconductors^4^ and electron-field emitters^2^. Fluorescent nanodiamonds have been used as non-toxic alternatives to semiconductor quantum dots for biomedical imaging^5^. Biocompatibility and resistivity enable use of nanodiamonds for coatings on medical implants^6^. The broad spectrum of possible functional chemical groups, biocompatibility, small sizes, and homogeneous size distributions offer extensive possibilities for using them in nanofabrication, bioconjugation, and drug delivery^3^,^7^.

Literature reports indicate that many diamond nanocrystals are structurally inhomogeneous, consisting of ordinary cubic (c-) diamond (space group: Fd-3m), plus several poorly characterized sp^3^-bonded forms, variously called polymorphs, allotropes, or polytypes^8^,^9^,^10^,^11^,^12^,^13^,^14^,^15^,^16^,^17^,^18^. For uniformity, we call these polymorphs. They are of interest to materials scientists because they are thought to possess exceptional physical properties. For example, h-diamond (aka lonsdaleite), whose existence as a distinct material was recently questioned^19^, has received attention because it is thought to possess superior compressive strength, hardness, and rigidity, compared to c-diamond^20^,^21^. The i-diamond polymorph has been proposed to be a super-dense form, with a calculated density of 4.1 g/cm3, which is 15% greater than that of c-diamond^22^. m-diamond was attributed to a material that could possess extreme hardness^23^. Wen *et al.*^24^ suggested that, in distinction to c-diamond, n-diamond absorbs electromagnetic radiation and thus could eliminate electromagnetic interference caused by wireless communication tools.

Nanodiamonds of extraterrestrial origin are abundant in some meteorites^25^,^26^,^27^ and have been reported from impact-related horizons in sedimentary rocks such as the K/Pg boundary^28^. Meteoritic nanodiamonds also have the potential to provide insights into early Solar System formation conditions^26^. Of particular interest are reports of a world-wide nanodiamond-rich layer associated with the onset of the Younger Dryas (YD) cooling episode at ~12,800 before present^29^,^30^,^31^,^32^,^33^. These nanodiamonds are postulated to have formed from terrestrial carbon during an extraterrestrial impact, producing c-, h-, i-, and n-diamonds^33^. According to this scenario, the catastrophic event had serious consequences on the climate and brought about the extinctions of most Pleistocene megafauna including Mammoths^29^,^30^,^31^,^32^,^33^. However, questions have been raised about the temporal association of nanodiamonds with the YD^34^,^35^, the occurrence of diamond polymorphs in these sediments^34^,^36^,^37^,^38^, and even evidence for impact signatures during the YD^39^. Although controversial, YD reports commonly use the presence of nanodiamond polymorphs as the impact signature.

The proposed polymorphs could be attractive for a range of applications and as proxies of major terrestrial impacts, but they have not been synthesized in pure form and have only been reported as mixtures of different carbonaceous phases such as graphite and amorphous carbon. As such, the structures and characteristics of the reported polymorphs are either controversial or not diagnostic (Table 1). In particular, the existence of h-diamond was recently questioned^19^, and several structural and chemical models were proposed for i-, m-, and n-diamond, including substitution of carbon by hydrogen^9^,^13^,^40^ and incompletely described diamond forms^41^. Recently, Li *et al*.^42^ suggested a structure model for n-diamond consisting of layers of h-diamond and *C*3 isosceles triangle rings. In any case, there is no consensus on the structure or synthesis conditions of the polymorphs.

Transmission electron microscopy (TEM) is uniquely suited for the structural and chemical investigation of individual nanosized particles. Diamond polymorphs have been reported from high-resolution transmission electron microscope (HRTEM) images and selected-area electron diffraction patterns based on features absent from c-diamond. However, these features are not unique for the presumed polymorphs (Supplementary Note 1, Supplementary Figs 1-2). We show that natural and synthetic c-nanodiamonds commonly consist of sub-nanometer twin domains, which give rise to the characteristic features attributed to the proposed diamond polymorphs.

## Results

### Nano-sized twins in c-diamond

In order to investigate the diamond-polymorph issue, we selected several synthetic and natural samples for study. The synthetic samples were produced through chemical vapor deposition (CVD), and the natural ones were obtained from primitive (Murchison and Orgueil) and impact-shocked (Gujba) meteorites^42^. Samples of similar origins reportedly contain various diamond polymorphs^9^,^10^,^11^,^12^,^13^,^16^,^25^,^27^.

**Figure 1:**
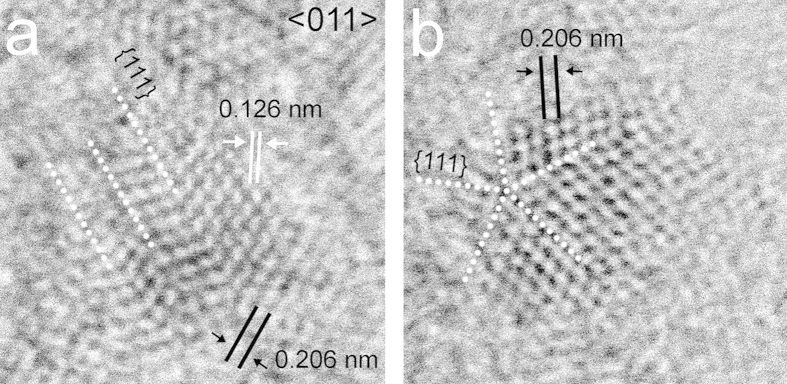
Twinned nanodiamonds from the Murchison and Orgueil meteorites. (**a**) Grain with multiple {111} twins along <011>. (**b**) Grain with <011> rotation twins exhibiting pentagonal symmetry. White dotted lines indicate the {111} twin planes.

**Figure 2:**
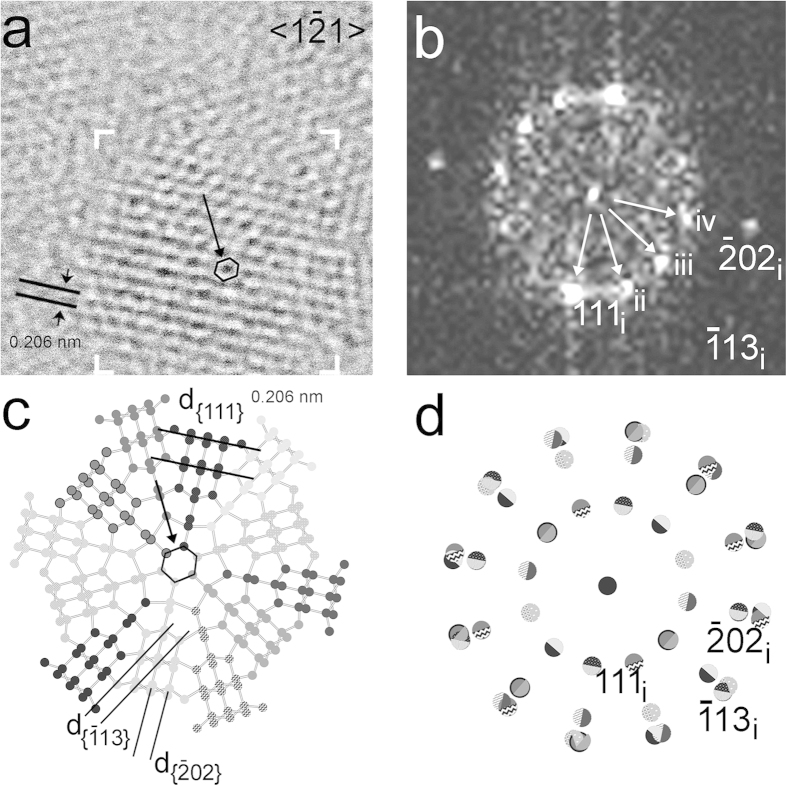
Nanodiamond grain exhibiting dodecagonal symmetry. (**a)** HRTEM image along <1
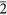
1> Nanodiamond grain from the Orgueil meteorite exhibiting dodecagonal symmetry. Black arrow points towards a hexagonal ring of carbon atoms (black). (**b**) FFT calculated from the area marked by white corners in (**a**) shows multiple sets of 0.206-nm {111} diffraction spots (white arrows). We interpret these spots as evidence for twin domains that are rotated around <1
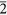
1> by 30°, i.e., for a grain displaying dodecagonal symmetry. Domains with different sizes account for the uneven intensity distribution of diffraction spots (cf. the intensities of the 111 diffraction spot for domains i, ii, iii, iv). (**c**) Structure model, deduced from the FFT, of an idealized 30° rotation twin containing 12 equal-sized domains (<1
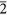
1> projection). The interface of the twin domains consists of five- and six-member carbon rings (the central ring is indicated by a black arrow). **(d)** Sketch of the diffraction pattern of the 30° rotation twins. Domains and their corresponding diffraction spots are illustrated by the balls having different patterns. The grain is a new example of non-crystallographic symmetry in nanoparticles.

**Figure 3:**
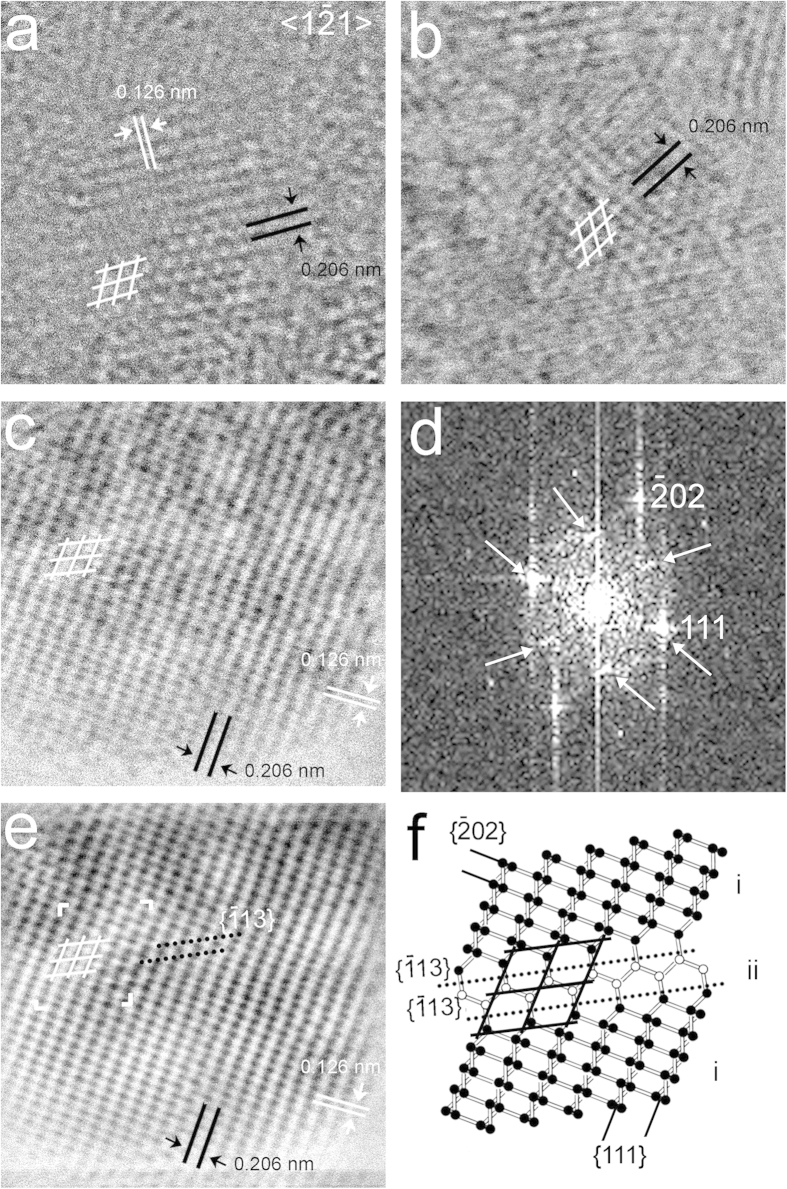
Nanodiamonds with hexagonal 0.206-nm fringes. (**a**) Grain from the Orgueil meteorite. (**b**,**c**) Grains from the Murchison meteorites. These grains contain sub-nanometer regions that exhibit hexagonal fringes when viewed along <1
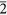
1> (white rhombuses). (**d**) FFT calculated from (**c**) shows hexagonally arranged diffraction spots (white arrows). (**e**) Background-filtered image calculated from (**c**). (**f**) Structure model of the region marked with white corners in (**e**). The model consists of large regions (domain i: black balls) with rectangular {111} and {202} cross-fringes and a small region (domain ii: white balls) with hexagonally arranged {111} fringes (black lines) resulting from two adjacent {113} twins (black dotted lines). The hexagonally arranged carbon atoms give rise to the hexagonally arranged diffraction spots. The intensity of these spots is a function of the width of the area with hexagonally arranged carbon atoms; i.e., layers of domain ii can be coherent with other ii layers even if separated by i layers, with a resulting increase in diffraction intensity.

**Figure 4:**
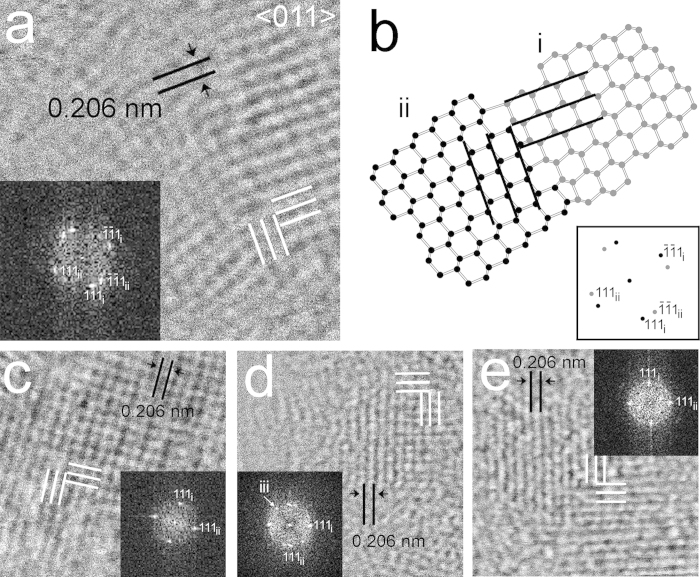
Diamond nanocrystals with rectangular 0.206-nm fringes (white lines). (**a**) HRTEM image of a grain from the CVD-produced sample. Corresponding FFT (insert) shows two sets of 0.206-nm {111} diffraction spots that indicate domains (i, ii) that are rotated 90° with respect to each other along <011>. (**b**) Structure model showing two domains of a 90º rotation twin. Black lines mark perpendicular {111} planes. The domain boundaries of the twin structure consist of five-, six-, and seven-member carbon rings. The sketch of the diffraction pattern of the 90° rotation twin (insert) shows two sets of {111} diffraction spots. Domain i and ii as well as their corresponding diffraction spots are marked with grey and black balls, respectively. (**c**) Grain from the CVD-produced sample (**d**,**e**) Grains from the the Orgueil meteorite. Domain misorientation (tilt from <011> zone axis) results in one set of 111 diffraction spots in FFTs (**c**,**d**,**e**). The FFT suggests that (**d**) consists of three domains (white arrow) from which two are related to 90° rotation.

**Figure 5:**
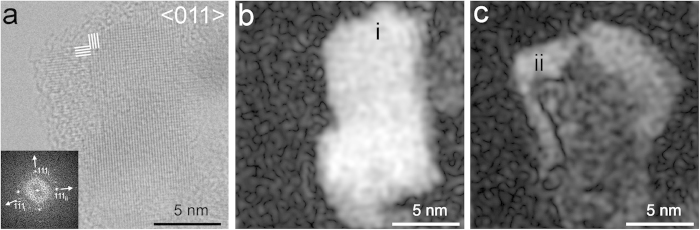
Rectangular 0.206-nm fringes (white lines) of a diamond nanocrystal from the Gujba sample and its interpretation with domains that are rotated 90° with respect to each other. (**a**) HRTEM image and its corresponding FFT (bottom left). (**b**,**c**) Amplitude images calculated from the 111 set of diffraction spots of the FFT. Bright areas correspond to twin domains i and ii, respectively.

HRTEM images of these nanodiamonds reveal fringes with 0.206-, 0.126-, and 0.108-nm spacings, corresponding to c-diamond {111}, {202}, and {113} planes, respectively (Figs 1 and 2). All investigated grains show one or more of these sets of fringes, and the C K-edge EELS spectral shapes from these crystals are consistent with c-diamond (Supplementary Fig. 2a). Twinning is characteristic of the nanocrystals (Figs 1, 2, 3, 4, 5), which commonly display both reflection and rotation twins. The most abundant types are the {111} reflection twins (Fig. 1a,b), also called ∑3 twins, which are consistent with previous studies^25^,^44^,^45^. These twins can give rise to the common <011> rotation twins^25^,^45^, in which the domains are rotated ∼71º with respect to each other around <011> (Fig. 1b). We report a new type of rotation twins (<1
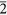
1> twins) (Fig. 2), which divide the grains into sub-nanometer domains, similar to the <011> rotation twins. The symmetry of these domains matches that of c-diamond. However, as a result of rotational twinning, the HRTEM images exhibit new non-crystallographic symmetry elements. For example, the grains in Figs 1b and 2a have pentagonal and dodecagonal symmetries. Pentagonal and decagonal symmetries are common in gold nanoparticles^46^,^47^, and five-fold twins occur in nanodiamonds^25^,^45^,^48^. The nanodiamond grain with dodecagonal symmetry (Fig. 2), in which the domains are rotated 30° to each other, is a new example of non-crystallographic symmetry in nanoparticles.

Grains exhibiting hexagonal- and square-fringe patterns having 0.206-nm spacings corresponding to c-diamond {111} planes (Figs 3, 4, 5) are prominent in each sample. The fast Fourier transforms (FFTs) calculated from these patterns show c-diamond diffraction spots that are related through two types of twins. The diffraction spots calculated from the hexagonal-fringe pattern (Fig. 3d) can be interpreted as arising from {113} reflection twins. This type of twin is especially abundant in the shock-formed Canyon Diablo diamond and in synthetic samples prepared under conditions in which lonsdaleite was reported^19^. The FFTs calculated from the square-fringe pattern exhibit rows of perpendicular diffraction spots with 0.206-nm spacings corresponding to c-diamond {111} planes and additional diffraction spots with the same spacings (Figs 4, 5a). This diffraction-spot arrangement is consistent with <011> rotation twins, in which the twin domains are rotated 90° with respect to each other around <011>. In order to clarify details of this twin type, we modeled its structure (Fig. 4b) and generated amplitude images from the {111} diamond diffraction spots (Fig. 5b,c). The intensity maxima of these images occur where the selected sets of fringes occur. They show the {111} lattice fringes occur in different regions and do not overlap completely (Fig. 5b,c), as is characteristic of twin domains. Thus, although the hexagonal and square-fringe patterns exhibit symmetries not expected for c-diamond, twinned c-diamond provides an explanation.

### Explanation for the diffraction features of the polymorphs

The twinned nanodiamonds described above display HRTEM images and spacings that match those used to recognize the reported diamond polymorphs. For example, nanosized grains of h-diamond have been identified from HRTEM images with hexagonal and square fringe-patterns^25^,^27^,^29^,^30^,^31^,^32^,^33^ showing 0.21-nm spacings. However, simulations of HRTEM images at different experimental conditions suggest the observed patterns are incompatible with this interpretation (Supplementary Fig. 3). The reported hexagonal and square-fringe patterns match those of Figs 3, 4, 5, i.e., they are consistent with the {113} twins and <011> rotation twins mentioned above. As such, the structural features attributed to h-diamond can be explained by twinned c-diamond. The case for i-diamond is more problematic as there is no consensus regarding its structure, and reports of its unit-cell dimension range between 0.250 and 0.428 nm, with primitive or i-centered symmetry (Table 1). Assuming the most commonly reported^9^,^18^,^22^ unit-cell parameter of 0.428 nm and i-centering with an *a* glide^22^, the HRTEM image of i-diamond matches the square fringe-pattern having 0.21-nm spacings. However, our analysis shows this pattern is consistent with <011> rotation twins of c-diamond. The proposed m-diamond, which was recently reported from HRTEM images and corresponding FFTs based on a 0.63-nm d-spacing^23^, can be explained by {111} c-diamond twins (Supplementary Fig. 4). These twins in thick crystals (electron is scattered more than 1) can give rise to the spacings and diffraction spots attributed to m-diamond. Therefore, the diffraction patterns and HRTEM images used to identify h-, i-, and m-diamond are compatible with twinned c-diamond.

The issue of n-diamond is ambiguous because its diagnostic d-spacings are not unique (Supplementary Fig. 1). For example, it is commonly identified using the 0.178-nm spacing (assigned as the 200 diffraction spots) from HRTEM images and their corresponding FFTs^10^,^11^,^12^,^13^,^17^,^30^,^31^,^32^,^33^. However, diffraction from dynamically scattered electrons for c-diamond yields the same spacing. Images show the intensity of the 200 diffraction spot, which violates the crystallographic selection rules for Fd-3m symmetry, arises with increasing sample thickness even in nanosized crystals (Supplementary Fig. 5). Identification from SAED ring patterns is similarly nondiagnostic because they are from areas large enough (>20-nm wide) to include grains of other nanomaterials. For example, graphite, whose d-spacings closely match those of n-diamond, is a common byproduct of synthesis and also occurs in natural samples together with c-diamond nanocrystals.

## Conclusions

The literature contains numerous reports of diamond polymorphs, including h-, i-, m-, and n- diamond^8^,^9^,^10^,^11^,^12^,^13^,^14^,^15^,^16^,^17^,^18^,^23^,^25^,^27^,^28^,^29^,^30^,^31^,^32^,^33^,^35^,^49^. However our ultra-high-resolution TEM data show that nanodiamonds are intimately twinned, and these defects give rise to the image and diffraction features used to identify h-, i-, m-, and n- diamond. This interpretation calls for reevaluation of previous reports and leads to a rethinking of materials and planetary science implications based on these polymorphs. For example, the reported exceptional material properties^10^,^18^,^19^,^20^,^23^ attributed to these polymorphs need to be reexamined. Furthermore, since nanosized diamond polymorphs reported in^28^,^29^,^30^,^31^,^32^,^33^ are not necessarily a marker for cosmic impact, and therefore inappropriate for inferring the YD impact origin, the question of that origin is worth reconsidering as also proposed in^34^,^36^,^37^,^38^,^39^.

Twinning is common in c-diamond nanocrystals, produces grains with intricate patterns of structure fringes in HRTEM images, and results in nanometer to sub-nanometer domains. Of the samples we studied, the twin domains are largest (>5 nm wide) in the Gujba meteorite, in which the crystals are also large (>10 nm wide) and which were reported to form as a result of shock metamorphism^43^. In contrast, the small twin domains (<1 nm wide) of Murchison and Orgueil are comparable in size to those of the CVD-produced diamond. Our findings imply that defective structure is favored for 1- to 5-nm diamond crystals, many of which contain abundant twin boundaries. These boundaries affect the mechanical, electronic, and optical properties of c-nanodiamonds and promote the implantation of foreign elements (dopants), which are used to prepare semiconductors, electron-field emitters and quantum dots^2^,^4^,^5^.

## Methods

### Materials and Methods

Small droplets (ca. 2 ml) of Gujba, Murchison, and Orgueil nanodiamond residues in suspension with water were dried on Cu grids coated with lacy-C. We also prepared TEM samples from (1) synthetic material provided by Andriy Sherehiy and Mahendra Sunkara (University of Louisville), grown in a chamber that was inadvertently contaminated with Cu; and (2) CVD-produced nanodiamonds provided by Robert Nemanich (Arizona State University). TEM data were acquired from electron-transparent areas of the residues protruding into the holes of the carbon-support film. HRTEM and bright-field STEM images were acquired with a JEOL 4000EX TEM (400 keV, 0.17-nm point resolution) and a JEOL ARM200F aberration-corrected scanning TEM (200 keV, 0.08-nm point resolution), respectively.

FFTs obtained from the HRTEM images were calculated using Gatan Digital Micrograph (DM) 3.5 software. The amplitude images of Fig. 5 were generated following the method described in^50^,^51^, using routines written for DM software and applying 0.2 nm^−1^ Lorentzian masks for the {−111} set of diamond diffraction spots. We performed this analysis on the Gujba sample, where the domains were sufficiently large (>5 nm wide). We set the upper and lower contrast limits to the same values for both images.

## Additional Information

**How to cite this article**: Németh, P. *et al.* Twinning of cubic diamond explains reported nanodiamond polymorphs. *Sci. Rep.* **5**, 18381; doi: 10.1038/srep18381 (2015).

## Supplementary Material

Supplementary Information

## Figures and Tables

**Table 1:** Diamond and reported nanosized polymorphs.

Name	Cell parameters (nm)	Space group	Diagnostic *d* spacings (in nm)***
**diamond**	a = 0.356	Fd-3m	0.206, 0.126, 0.107
**h-diamond**	a = 0.252 c = 0.412	P6_3_/mmc^52^	**0.218**, 0.206, **0.193**, **0.150**, 0.126, **0.116**, **0.109**, 0.107
**n-diamond**	0.356*	Fm-3m^49^, R-3m^53^, P4_2_/mmc^11^	0.206, **0.178**, 0.126, 0.107, **0.103**
**i-diamond bcc phase**^9^ **0.25-i-carbon**^49^	0.428** 0.31^9^ 0.25^49^	Im-3^18^, Ia-3^22^, P4_2_/mmc^11^, P2_1_3^12^, P4_2_3_2_^12^	**0.303, 0.214**, **0.175**, **0.152**,**0.135**, 0.124, **0.114**, 0.101****
**m-diamond**^23^	a = 0.436, b = 0.251, c = 1.248, b = 90.9°^23^	Unknown	**0.624, 0.312, 0.218, 0.214, 0.208,** 0.207**, 0.205, 0.180, 0177, 0.165, 0.151, 0.149,** 0.1278**, 0.109, 0.108,** 0.107

Comments:

*Face-centered cubic metals used for TEM grids and in equipment for c-diamond syntheses have almost identical cell dimensions. In particular, Cu was reported from TEM samples containing the YD material^36^, and we encountered it in a synthetic sample designed to produce n-diamond (Supplementary Fig. 1).

**Vora and Moravec^54^ reported its cell parameter between 0.396 and 0.428 nm.

***Reflections absent in Fd-3m diamond are in bold.

****d-spacings calculated for Im-3 space group with a = 0.428 nm cell dimension.
