# Supplementary material for: Twinning of cubic diamond explains reported nanodiamond polymorphs
Source: Sci Rep. 2015 Dec 16;5:18381. doi: 10.1038/srep18381 (PMC4680963; doi:10.1038/srep18381)
Supplement: Supplementary Information [file srep18381-s1.pdf]

Supplementary Information:

Twinning of cubic diamond explains reported nanodiamond polymorphs

Péter Németh[1], Laurence A.J. Garvie[2,3], and Peter R. Buseck[3, 4]

[1]Institute of Materials and Environmental Chemistry, Research Center for Natural Sciences, Hungarian Academy of Sciences, H-1117 Budapest, Magyar Tudósok Körútja 2, Hungary.

[2]Center for Meteorite Studies, Arizona State University, Tempe, Arizona 85287-6004, USA.

[3]School of Earth and Space Exploration, Arizona State University, Tempe, Arizona 85287-6004, USA.

[4]School of Molecular Sciences, Arizona State University, Tempe, AZ 85287-1604, USA.

*Correspondence to: nemeth.peter@ttk.mta.hu

Supplementary Note 1

d-spacing measurements and presumed diamond polymorphs

The presumed diamond polymorphs were characterized in the literature by their symmetries and cell parameters (Table 1). However, serious challenges exist in making measurements to the required accuracy to distinguish among the characteristic d-spacings. Even well-calibrated HRTEM images, obtained by using a well-aligned microscope, show errors of ±2%[supplementary reference 1]. Thus, it is not normally possible to distinguish between the 0.214-nm spacing (002) of i-diamond and the 0.206-nm spacing (111) of Fd-3m diamond and between the 0.218 nm (100) of h-diamond and 0.212 nm (100) of graphite. Thus, the interpretation of diamond polymorphs based on these d-spacings [supplementary reference 2-11] is inconclusive. Although powder X-ray diffraction has also been used for identification (e.g., [supplementary reference 5, 11]), it can not be used for characterizing individual grains.

Supplementary Figures

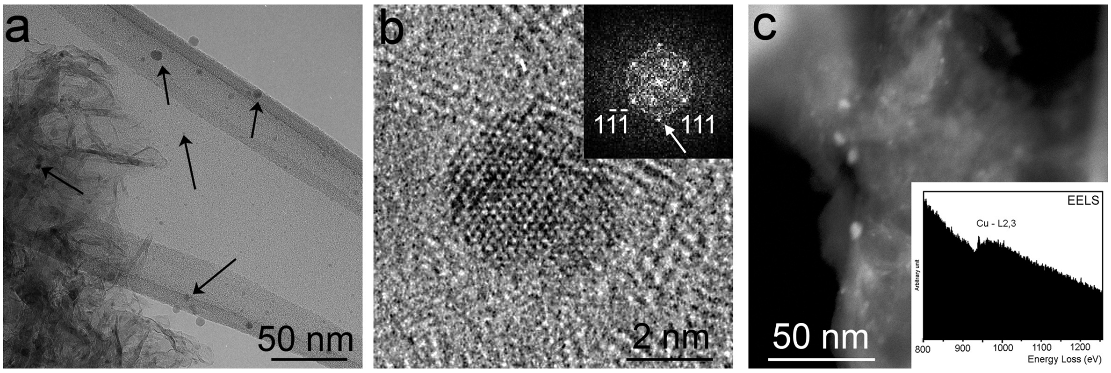

Supplementary Figure 1: The features of nanocrystalline Cu could be confused for n-diamond. a) Bright-field TEM image from a synthetic sample originally thought to contain n-diamond. Nanocrystals (arrows) have high contrast, similar to those reported in [supplementary reference 2-11], relative to the carbonaceous matrix and the lacey-carbon grid. **b)** HRTEM image of a nanosized Cu particle. The FFT (top-right corner) of the image is indexed according to Cu along <110>. A white arrow shows the 200 reflection of Cu, which can be mistakenly identified as 002 of n-diamond. **c)** The particles occur as bright spots in a carbonaceous matrix on the dark-field STEM image, similar to those reported in [supplementary reference 10-11]. The EELS data of the grain (bottom-right corner) indicates Cu.

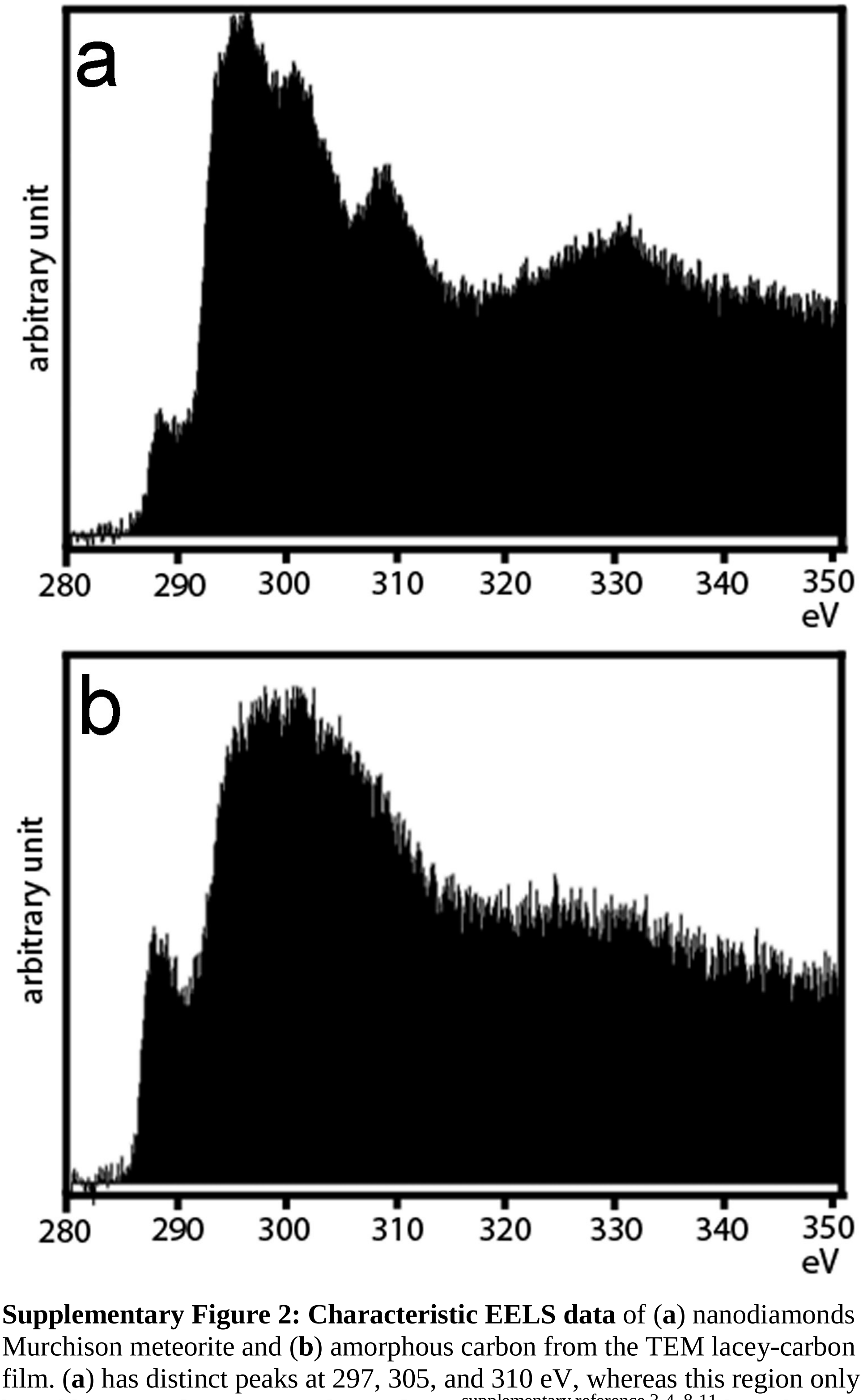

Supplementary Figure 2: Characteristic EELS data of (**a**) nanodiamonds from the Murchison meteorite and (**b**) amorphous carbon from the TEM lacey-carbon support film. (**a**) has distinct peaks at 297, 305, and 310 eV, whereas this region only displays a broad hump in (**b**). Data similar to (**b**) in [supplementary reference 3-4, 8-11] have been attributed to diamond polymorphs.

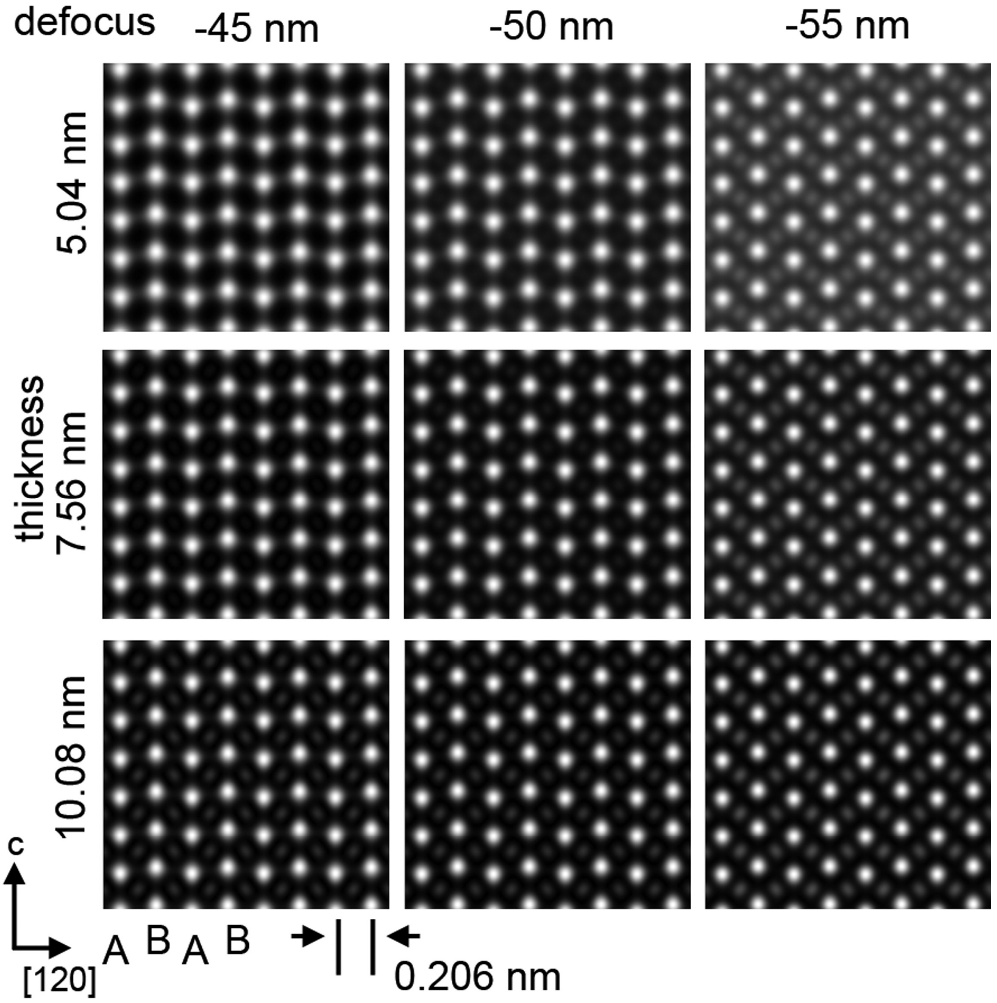

Supplementary Figure 3: Simulated HRTEM images of the supposed h-diamond polymorph along <100> at various values of thickness and defocus. The images are similar to each other in that they show a subtle zig-zag pattern along [120] corresponding to AB stacking. This pattern does not occur on the images of Figs. 4-5 and in [supplementary reference 1, 12], and thus they are inconsistent with the interpretation of h-diamond. The simulations were performed with the JEMS software [supplementary reference 13] using the multislice method, the structure data reported by [supplementary reference 14], and applying the microscope parameters for a JEOLARM TEM (accelerating voltage: 200kV, spherical aberration coefficient: 0.1 mm)

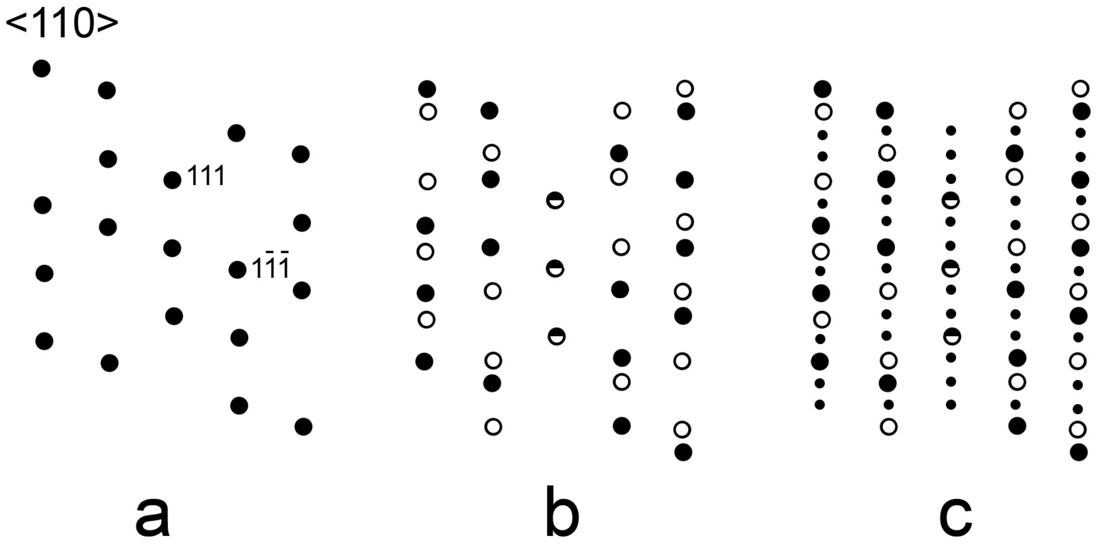

Supplementary Figure 4: Twins and sample thickness give rise to the 0.63-nm diffraction maxima attributed to m-diamond. a) Reciprocal lattice of single-crystal diamond (black balls). The 111 reflections have 0.206-nm spacings. **b)** Reciprocal lattice of {111} twinned diamond (white balls). **c**) Reciprocal lattice of {111} twinned diamond from a thick crystal (electron is scattered >1). Reflections with ~0.63-nm (triple 0.206 nm) spacings arise from multiple scattering (small black spots). Patterns similar to (**c**) were reported for twinned crystals [supplementary reference 15] and were mistakenly attributed to m-diamond [supplementary reference 16].

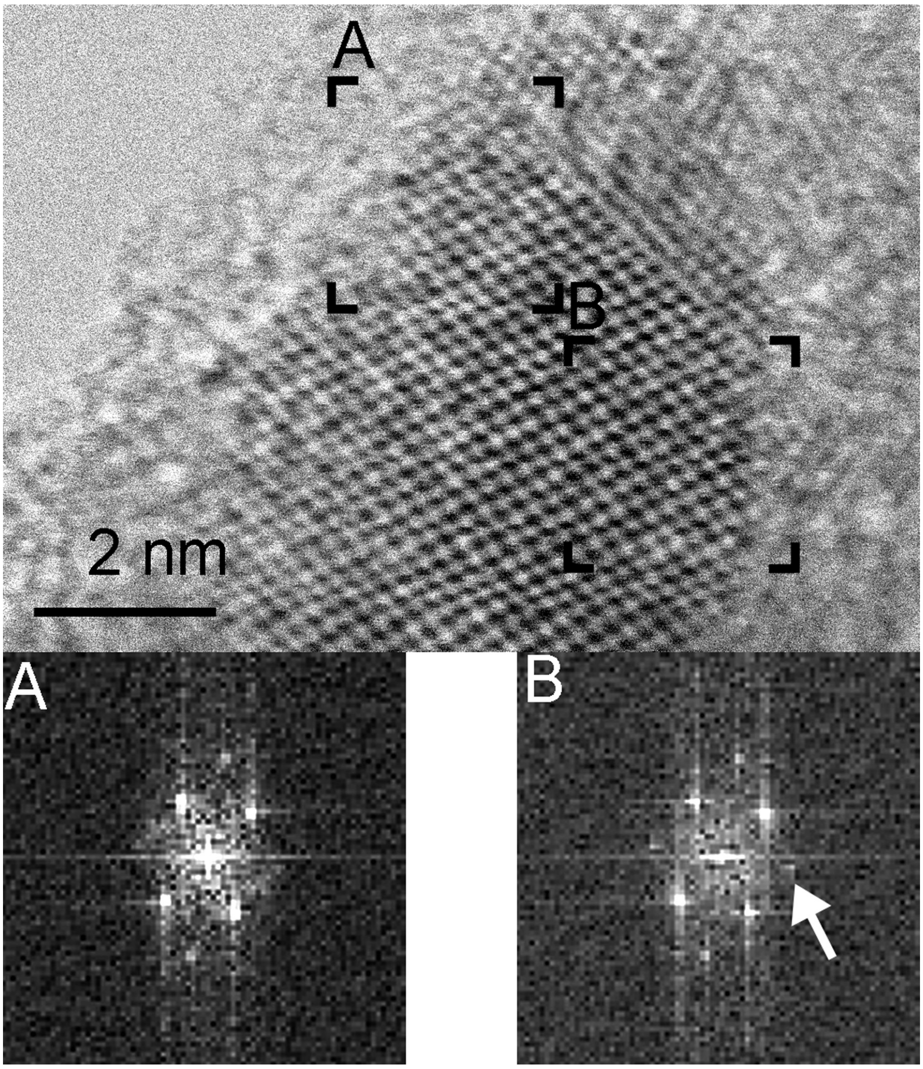

Supplementary Figure 5: Sample thickness explains the diagnostic features of n-diamond. HRTEM image and corresponding FFTs of a grain from the CVD nanodiamond. The thin part of the grain (area A), close to the hole (left upper side of the image), displays the diffraction pattern expected for Fd-3m symmetry, whereas sample thickness gives rise to the 200 reflection of c-diamond (white arrow), which has been mistakenly attributed to n-diamond. Black line marks 2 nm.

Supplementary References

1. Daulton, T. L., Eisenhour, D. D., Bernatowicz, T. J., Lewis, R. S. & Buseck, P. R. Genesis of presolar diamonds: comparative high-resolution transmission electron microscopy study of meteoritic and terrestrial nano-diamonds. *Geochim. Cosmochim. Acta* **60**, 4853-4872 (1996).
2. Bucknum, M.J. & Castro, E. A. On the n-diamond and i-carbon nanocrystalline forms. *J. Math. Chem.* **50**, 1034-1038 (2012).
3. Peng, J.L., Orwa, J.O., Jiang, B., Prawer, S. & Bursill, L.A. Nano-crystals of c-diamond, n-diamond and i-carbon grown in carbon-ion implanted fused quartz. *Int. J. Mod. Phys. B* **15**, 3107–3123 (2001).
4. Konyashin, I. *et al.* A new hard allotropic form of carbon: Dream or reality? *Int J Refract Metals Hard Mater* **24**, 17–23 (2006).
5. Kumar, A. *et al.* Formation of nanodiamonds at near-ambient conditions via microplasma dissociation of ethanol vapour. *Nat. Comms.* DOI: 10.1038/ncomms3618 (2013).
6. Kennett, D. J. *et al.* Nanodiamonds in the Younger Dryas boundary sediment layer. *Science* **323**(5910), 94-94 (2009a).
7. Kennett, D. J. *et al.* Shock-synthesized hexagonal diamonds in Younger Dryas boundary sediments. *Proc. Natl. Acad. Sci. U.S.A.* **106**(31), 12623-12628 (2009b).
8. Kurbatov, A. V. *et al.* Discovery of a nanodiamond-rich layer in the Greenland ice sheet. *Journal of Glaciology* **56**(199), 747-757 (2010).
9. Israde-Alcantara, I. *et al.* Evidence from central Mexico supporting the Younger Dryas extraterrestrial impact hypothesis. *Proc. Natl. Acad. Sci. U.S.A.* **109**(13), E738-E747 (2012).
10. Kinzie, C. R. *et al.* Nanodiamond-Rich Layer across Three Continents Consistent with Major Cosmic Impact at 12,800 Cal BP. *J. Glaciol.* **122**(5), 475-505 (2014).
11. Bement, L. C. *et al.* Quantifying the distribution in the pre-Younger Dryas to recent age deposits along Bull Creek, Oklahoma Panhandle, USA. *Proc. Natl. Acad. Sci. U.S.A.* **111**(5), 1726-1731 (2014).
12. Garvie, L. A. J. Surface electronic structure of meteoritic nanodiamonds. *Meteorit. Planet. Sci.* **41**(5), 667-672 (2006).
13. Stadelmann, P. A. EMS - A Software Package for Electron-Diffraction Analysis and HREM Image Simulation in Materials Science. *Ultramicroscopy* **21**(2), 131-146 (1987).
14. Bundy, F. P. & Casper, J. S. (1967) Hexagonal diamond - A new form of carbon. *J. Chem. Phys.* **46**, 3437-3446.
15. Huang, Q. *et al.* (2014) Nanotwinned diamond with unprecedented hardness and stability. *Nature* **510**, 250-253.
16. Daulton, T. L. *et al.* Polytype distribution of circumstellar silicon carbide: Microstructural characterization by transmission electron microscopy. *Geochim. Cosmochim. Acta* **67**, 4743-4767 (2003).
